# Semi-Empirical Model of Remote-Sensing Reflectance for Chosen Areas of the Southern Baltic

**DOI:** 10.3390/s22031105

**Published:** 2022-02-01

**Authors:** Barbara Lednicka, Maria Kubacka

**Affiliations:** 1 Department of Environmental Protection, Maritime Institute, Gdynia Maritime University, ul. Trzy Lipy 3, 80-172 Gdańsk, Poland; blednicka@im.umg.edu.pl; 2 Department of Operational Oceanography, Maritime Institute, Gdynia Maritime University, ul. Długi Targ 41/42, 80-830 Gdańsk, Poland

**Keywords:** remote-sensing reflectance, inherent optical properties, bio-optical algorithms, suspended and dissolved matter, biomonitoring, coastal waters

## Abstract

Coastal waters are the richest parts of ocean ecosystems characterised by dynamic changes in water biology, physical and chemical features. Establishing local relationships between water constituents and optical properties in these areas will help to develop successful ocean colour algorithms allowing a thorough understanding of complex coastal waters and improving water quality monitoring. In this paper, the authors present the use of optical and biogeochemical measurements in complex aquatic environments and aim to create a semi-empirical model of remote-sensing reflectance (*R_rs_*(*λ_i_*)) for four wavelengths (*λ_i_* = 420 nm, 488 nm, 555 nm, and 620 nm) based on multiparameter algorithms of absorption (*a*(*λ_i_*)) and backscattering (*b_b_*(*λ_i_*)) coefficients. The bio-optical properties of water were determined based on empirical data gathered from aboard the r/v Oceania from April 2007 to March 2010 in chosen areas of the southern Baltic (Polish coast). The analyses reveal that *R_rs_*(*λ_i_*) in the studied area can be described with satisfactory accuracy using a five-parameter model. Positive results with a statistical error magnitude of *R_rs_*(*λ_i_*) of less than 50% were achieved for all four applied wavelengths. Bio-optical algorithms proposed by the authors enable evaluating biogeochemical characteristics of coastal areas in a broader context of ecosystem assessment and contribute significantly to the development of Earth and environmental sciences.

## 1. Introduction

### 1.1. Context of the Study

The remote-sensing reflectance (*R_rs_*) is a crucial parameter in optical oceanography and is often used for the development of algorithms to estimate bio-optical components of seawater. Diffusion of light upon interaction with matter depends on the types and concentrations of the water components. Suspended and dissolved matter changes the optical properties of water, particularly in bays and coastal areas. The coastal waters of the Gulf of Gdańsk (the southern part of the Baltic Sea, Poland) are areas of high diversity and dynamic changes in physical and chemical properties of water, making this area an ideal location to study optical properties of water. As a typical coastal environment, these waters are characterised by specific physical conditions such as limited light penetration and high rates of transport and sedimentation of organic suspended particulate matter (*SPM_org_*) and inorganic suspended particulate matter (*SPM_inorg_*). There are many algorithms for estimating surface concentrations of suspended particulate matter (*SPM*) based on remote-sensing measurements [1,2]. However, most of them have been developed for the so-called “Case 1 waters” [3], i.e., areas where phytoplankton is the main factor responsible for variations in optical properties of the water (mostly open oceans) [4]. On the other hand, coastal waters and bays known as Case 2 waters [3] are influenced not just by phytoplankton but also by other substances that vary independently of phytoplankton *SPM_inorg_* and coloured dissolved organic matter (*CDOM*) [5,6,7]. Therefore, to apply remote sensing in such areas, their variability must be understood. The extraordinarily high optical diversity of Case 2 waters makes interpretation of optical signal from these waters rather difficult and more complex in terms of composition and optical properties than in Case 1 waters. The problem described also applies to the coastal waters of the Gulf of Gdańsk, where the correlation between optical properties and concentrations of optically active components is strictly local.

### 1.2. State of Knowledge

In recent years, there has been observed an intensive development of bio-optical models and algorithms describing the relationships between the optical properties of water and its composition. Methods for measuring radiation from different spectral ranges allowed developing many useful algorithms, which associate water properties with various physical, chemical, and biological processes [6,8,9,10,11,12]. For coastal ocean waters, internal seas (such as the Baltic Sea) and other waters classified as Case 2 waters (lakes, rivers), the correlations between optical properties and the surface concentrations of chlorophyll *a* (*Chl a*) are much weaker than in Case 1 waters and are often purely local. Consequently, the interpretation of optical signals from these sea areas classified as Case 2 waters presents far more difficulties. The main problem in developing algorithms for these waters is the complexity of their optical properties [13]. This happens because in such areas, there are many other biogeochemical components apart from autogenous ones (for example minerals and sediments), the concentrations of which correlate well with the content of *Chl a*. For example, the southeastern Baltic Sea is highly polluted with oil products [14]. The analysis based on the optical model of the Sea Basin identified the most universal spectral index of R_rs_ for 555 nm/440 nm for dispersed oil detection using any optical parameters [15]. The impact of absorption coefficient (*a*(*λ_i_*)) and backscattering coefficient *b_b_*(*λ_i_*) on *R_rs_*(*λ_i_*) is highly variable, thus the interpretation of reflectance spectra requires a simultaneous multi-parameter analysis of light propagation in seawater [16,17].

Over the last dozen years or so, the development of algorithms for Case 2 waters has been observed. However, these algorithms remain a niche problem. Semi-empirical algorithms are based on the primary theoretical physical relationships between the inherent optical properties (*IOPs*) (e.g., *a*(*λ_i_*) and *b_b_*(*λ_i_*)) and apparent optical properties (*AOPs*) (e.g., *R_rs_*(*λ_i_*)) and also statistical relationships between the *IOPs* and the biogeochemical components of water. Such algorithms are typically used to describe optically complex Case 2 waters [18,19,20,21,22,23,24,25]. However, because they show a very high sensitivity to changes in the composition and concentration of various water components, their range of applicability is limited and they can only be used locally.

Currently, attempts to model reflectance for remote sensing purposes focus mainly on the development of theoretical models in which reflectance is a function of *IOPs* [26,27] and the study of the relationship between the *IOPs* of seawater and types and concentrations of substances present in the water [25,28,29,30,31,32].

Studies of Case 2 waters conducted in recent years have revealed a very high need for local empirical and semi-empirical algorithms [2,11,31,32,33,34,35] which allow better estimation of components present in these waters and faster management response to possible pollution in the area. Since Case 2 waters are often strategically important regions (mainly coastal waters), it is extremely important to find the closest possible local relationships between the components of seawater and its optical properties. The innovativeness of our work is based not only on the technologically advanced and very rich survey methodology, but also on the locally-oriented approach to examining the optical properties of Case 2 waters.

### 1.3. Research Objectives

One of the current trends in optics of natural waters is to investigate the mutual relations between their *IOPs* and *AOPs*, as well as the relationships of these optical properties with the biogeochemical properties of water. Because *IOPs* are strongly related to the chemical composition of water, they may provide information on the concentrations and types of *SPM* and dissolved substances found in individual water bodies [25,31,32,36,37]. On the other hand, such a parameter as *R_rs_*(*λ_i_*), which is easily measurable and forms a basis for remote-sensing methods, is (under certain conditions of external lighting such as optical state of the atmosphere, cloud cover, position of the sun in the sky, etc.) closely related to *a*(*λ_i_*) and *b_b_*(*λ_i_*). Hence, the knowledge of all these interdependencies, especially the determination of their mathematical quantitative descriptions, is extremely important in the development of remote-sensing methods of controlling the condition and functioning of marine ecosystems (e.g., via satellites).

Considering the above we have focused on the following research objectives:

1. determination of a mathematical description of the relationship between the selected optical properties (absorption coefficient of phytoplankton (*a_ph_*(*λ_i_*)), absorption coefficient by non-algal particles (*a_d_*(*λ_i_*)), the coloured dissolved organic matter absorption coefficient (*a_CDOM_*(*λ_i_*)), backscattering coefficient of particles (*b_bp_*(*λ_i_*))) and the concentrations and physicochemical properties of natural water components (*Chl a*, *SPM*, surface concentrations of absorption coefficients of coloured dissolved organic matter for wavelength 400 nm (*a_CDOM_*(400)), sum of surface concentrations of accessory pigments (∑*C*), *SPM_inorg_*) in selected areas of the southern Baltic;
2. development of a semi-empirical model of *R_rs_*(*λ_i_*) enabling the determination of *R_rs_*(*λ_i_*) spectra in the visible light range based on the known spectra of *a*(*λ_i_*) and *b_b_*(*λ_i_*) in coastal waters of the southern Baltic or based on knowledge of the concentration of admixture components.

We expect that the obtained results can be used in remote-sensing and contribute significantly to the advancement of *b_b_* and particle size distribution in Case 2 waters.

## 2. Materials and Methods

### 2.1. The Conception of the Five-Parameter Model of R_rs_

**Figure 1:**
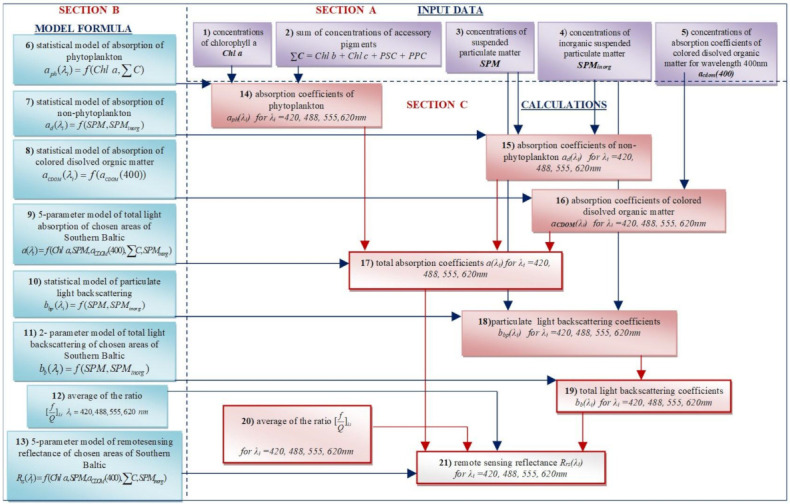
Block diagram of the five-parameter model of *R_rs_* in the selected areas of the southern Baltic for four wavelengths (*λ_i_* = 420 nm, 488 nm, 555 nm, and 620 nm).

To determine the relationship between water constituents and optical properties based on the in situ optical measurements and water sample analysis, first, we found a strictly local relationship between water constituents and optical properties in selected areas of the southern Baltic. In Figure 1 we present a block diagram of the proposed five-parameter model of *R_rs_* consisting of three sections: A—input data, B—model formula and C—calculations. Before starting work on the model, we checked the correlations between IOPs (*a_ph_*, *a_d_*, *a_CDOM_* and *b_bp_*) and the biogeochemical constituents (including organic fraction of *SPM_org_*). We chose the best correlations between biogeochemical components and optical properties for our work. This paper presents the strongest correlations between the aforementioned parameters.

For technical reasons (limitation of measuring equipment), our algorithms were developed for four wavelengths (*λ_i_* = 420 nm, 488 nm, 555 nm and 620 nm). First, regression methods of non-linear functions of one variable were used to designate 1-parameter statistical algorithms of *b_bp_*: *b_bp_*(*λ_i_*) = *f*(*SPM*). This allowed us to determine values of this optical parameter based on the predetermined surface concentrations of *SPM*. Similar one-parameter statistical analyses were conducted for other measured optical properties: *a_ph_*, *a_d_*, *a_CDOM_* (*a_ph_*(*λ_i_*) = *f*(*Chl a*), *a_d_*(*λ_i_*) = *f*(*SPM*), *a_CDOM_*: *a_CDOM_*(*λ_i_*) = *f*(*a_CDOM_*(400))) and concentrations of biogeochemical (and optical) constituents of waters-*Chl a*, *SPM*, *a_CDOM_*(400).

*a_CDOM_* is complex due to the variety of chemical structures of substances dissolved in natural waters and differentiated interaction of their molecules with electromagnetic radiation. They include both saturated substances (which practically do not absorb light in the uv visible spectrum) and strongly absorbant unsaturated substances. It would be pointless to search for any dependency between *a_CDOM_* and *SPM*. Therefore, the “optical” indicator of the concentrations of *CDOM* substances we adopted is *a_CDOM_* for the selected reference wavelength *a_CDOM_*(*λ_ref_*), which has been used commonly for many years now [4,31,37,38,39]. We assumed that *λ_ref_* = 400 nm, because *λ_ref_* is mostly located in the violet-blue region of the spectrum.

Next, we developed regional semi-analytic biogeochemical multiparameter algorithms to retrieve concentrations of seawater constituents and optical properties for these areas. Based on one-parameter statistical algorithms of chosen areas of the southern Baltic, we developed a multi-parameter algorithm e.g., for the *b_bp_*: *b_bp_* = *f*(*SPM*, *SPM_inorg_*), that allowed us to calculate the value of *b_bp_*, based on e.g., *SPM* and *SPM_inorg_*. Similar multi-parameter algorithms were employed for other optical properties and water constituents. We developed a statistical two-parameter model of *a_ph_*: *a_ph_*(*λ_i_*) = *f*(*Chl a*, ∑*C*) and a statistical two-parameter model of *a_d_*: *a_d_*(*λ_i_*) = *f*(*SPM*, *SPM_inorg_*). *a*(*λ_i_*) was calculated based on the equation [40]:

*a*(*λ_i_*) = *a_ph_*(*λ_i_*) + *a_d_*(*λ_i_*) + *a_CDOM_*(*λ_i_*) + *a_w_*(*λ_i_*), (1)

where *a_w_*(*λ_i_*) is the absorption coefficient of seawater molecules [41]. *b_b_*(*λ_i_*) was estimated as [40]:

*b_b_*(*λ_i_*) = *b_bp_*(*λ_i_*) + *b_bw_*(*λ_i_*), (2)

where *b_bw_*(*λ*) is the backscattering of seawater molecules according to Morel [42].

Next, we obtained (*f/Q*)_*i*_ values for the four tested wavelengths, *λ_i_* = 420, 488, 555 and 620 nm.

The final stage of the analysis was the construction of a semi-empirical five-parameter model of *R_rs_*: *R_rs_*(*λ_i_*) = *f*(*Chl a*, ∑*C*, *SPM*, *SPM_inorg_*, *a_CDOM_*(400)) for the selected areas of the southern Baltic, based on multi-parameter algorithms for *a*(*λ_i_*) and *b_b_*(*λ_i_*).

### 2.2. The Study Area

The Baltic is a sea with a constricted inflow of salty ocean waters as the straits connecting it with the North Sea are narrow and shallow. At the same time, the outflow of river- and rainwater remains large as the Baltic Sea collects water from 250 rivers, most of which are on the west (the Scandinavian Peninsula).

**Figure 2:**
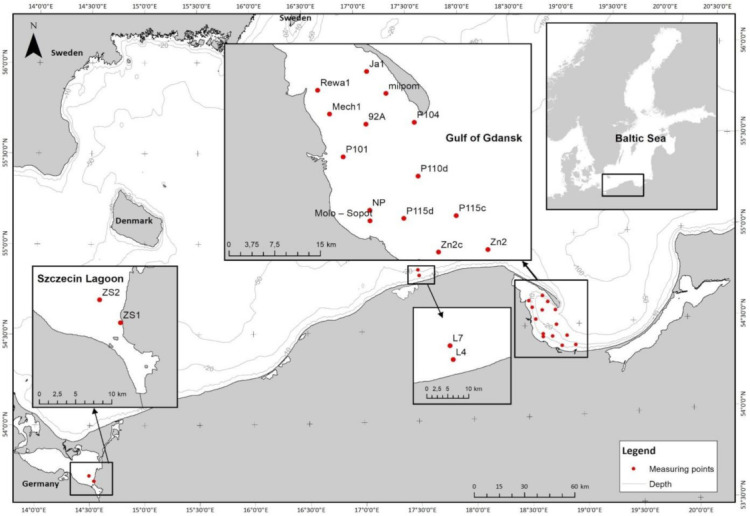
Locations of the study area and sampling stations.

The area where the measurements of selected *IOPs and AOPs* were carried out is in the coastal waters of the southern Baltic (Poland) (Figure 2), where a very large amount of pollution generated by agriculture and industry accumulates, reaching the sea in the form of sewage, or by atmospheric deposition. The most harmful substances are carried in by the rivers of the southern and eastern Baltic Sea, i.e., the Odra, Vistula, Dziwna, Niemen and Neva. The estuaries of these rivers are among the most polluted regions on the coast of the Baltic Sea [43]. Therefore, the coastal zone of the Baltic Sea is a “perfect laboratory” to study the impact of the concentrations of various admixtures on the optical properties of water. Additionally, the large variability of the physical and biological parameters of seawater can be observed in this zone due to local upwellings [44].

The measurement stations were located in the Gulf of Gdańsk, in the coastal zone of the southern Baltic, and the Szczecin Lagoon. The Gulf of Gdańsk (with 14 measurement stations) stands out against the background of the southern Baltic due to its fairly large depth and a gentle and even coastline dominated by flat and sandy beaches, with few steep slopes or cliffs. The seabed of the bay is mostly covered with fine-grained sand and silt. In the near-seabed zone, oxygen deficiency, high concentrations of hydrogen sulfide, and increased concentrations of heavy metals are very common [45]. This contamination is caused primarily by the pollution carried in by the waters of the Vistula and Pregoła rivers entering the bay. The western part of the Gulf of Gdańsk is Puck Bay, the waters of which are heavily polluted and characterised by very low salinity (even below 1‰) [46].

Two measurement stations are located in the coastal zone of the southern Baltic. Due to its location, the area is characterised also by high dynamics of water flow. This favours the mixing of water in vertical planes and exchange with waters of the southern Baltic. The Szczecin Lagoon (with two measurement stations) is connected with the Baltic Sea by straits: Piana in the west, Świna between the islands of Uznam and Wolin, and Głęboki Nurt and Dziwna in the east [47]. The mean depth of this area is approximately 4 m. Waters of the Szczecin Lagoon are classified as brackish waters, i.e., they are a mixture of fresh river waters and salty sea waters, the salinity of which is lower than that of sea waters but higher than that of river waters [48]. The salinity of this waterbody ranges from 0.5‰ to 2‰ [49]. Sometimes, the salinity in certain parts of the lagoon reaches even 6‰, which is caused by the inflow of water from Pomeranian Bay through the Świna Strait.

### 2.3. Data Acquisition and Processing

Surface water samples were collected in situ, onboard the r/v Oceania, from April 2006 to March 2009, from 18 measurement stations during nine sampling campaigns. Additionally, 16 measurements were carried out in the area of the pier in Sopot in 2006–2008 (the “Molo-Sopot” point). Water samples were collected for analyses using a Niskin water sampler, from just below the surface, at each station. At the same time, the *b_bp_*(*λ_i_*) were estimated based on in situ measurements in the near-surface layer (1 m depth) using a spectral backscattering meter Hydroscat-4 (HOBI Labs, Bellevue, Washington, WA, USA) at four wavelengths (*λ_i_* = 420, 488, 550 and 620 nm). Furthermore we used the radiometer Ocean Colour Profiler OCP–100 (Satlantic Inc., Victoria, BC, Canada) to estimate *R_rs_*(*λ_i_*) from the in situ measurements. Next, the values of *R_rs_*(*λ_i_*) calculated using only the OCP-100 were used to calculate the *f/Q* ratio.

Water samples were taken for the study of biogeochemical parameters, from which the concentrations of *Chl a*, *SPM*, *SPM_inorg_*, and ∑*C* were obtained, and the following optical parameters of the water were measured: *a_ph_*(*λ_i_*), *a_d_*(*λ_i_*), *a_CDOM_*(*λ_i_*). During vessel cruises filtration of water samples was conducted right after collection. In the research trips to the pier in Sopot, filtration was performed a few hours after the sampling.

The dry weight of *SPM*, *SPM_org_* and *SPM_inorg_* was calculated using the standard gravimetric technique [25,50,51]. The concentration of *SPM* was measured gravimetrically after filtration of the same amount of water through pre-weighed and pre-combusted filters; next, the inorganic fraction was weighed after combustion. The organic fraction was determined by subtracting *SPM_inorg_* from *SPM*.

Samples aimed for the determination of *Chl a* concentrations were obtained by filtration of the water sampled (from 0.5 to 5 litres) under a pressure not exceeding 0.4 atm, through Whatman GF/F filters with a diameter of 47 mm. Next, *Chl a* was isolated from phytoplankton cells in the process of 24 h extraction with an organic solvent −96% ethanol [52,53]. After that, the samples were centrifuged (4000× *g* rpm, 15 min) to remove debris and cell remains. Then, a spectrophotometric measurement was performed using the UNICAM UV-400 spectrophotometer. The spectrophotometric measurements provided information on the absorbance *A*, based on which the concentration of *Chl a* was calculated [53]. Other phytoplankton pigments were determined using high-performance liquid chromatography (HPLC) as described by Strickland and Parsons [54], Parsons et al. [55], and Mantoura and Repeta [56]. Pigments were extracted from phytoplankton cells through ultrasonically assisted mechanical homogenisation, using 90% aqueous acetone [54,55]. Pigment concentrations were determined based on “external standardisation” [56], which combines the parameters obtained in the chromatographic separation of dyes with parameters related to the sampling and extraction conditions.

The methodology of preparing water samples of *a_CDOM_*(*λ_i_*) and *a_CDOM_*(400) for spectrophotometric analysis used two-step filtration and appropriate storage of samples awaiting laboratory analysis (at 4 °C, for no longer than 3 weeks) [57,58]. The first step of filtration eliminates large particles of suspended matter. To this purpose, a GF/F filter (by Whatman, with a pore size of 0.7 μm) was applied. The second stage was the removal of the smallest suspended particles using cellulose membrane filters (Sartorius) (pore size 0.2 μm). The spectra of *a_CDOM_*(*λ_i_*) were measured with a spectrophotometer in a 10 cm cuvette relative to Milli-Q water using samples filtered through a pre-rinsed 0.2 μm filter [59]. The total particulate absorption −*a_p_*(*λ_i_*), was measured with a spectrometer employing the Whatman GF/F filter pad technique [60] followed by depigmentation with sodium hypochloride, which separates *a_ph_*(*λ_i_*) and *a_d_*(*λ_i_*) components. *a_ph_*(*λ_i_*) was calculated as the difference between *a_p_*(*λ_i_*) and *a_d_*(*λ_i_*):

*a_ph_*(*λ_i_*) = *a_p_*(*λ_i_*) − *a_d_*(*λ_i_*). (3)

Spectral *b_b_*(*λ_i_*) was measured in situ using backscattering meter Hydroscat-4 (HOBI Labs) at four different wavelengths: 420 nm, 488 nm, 555 nm, 620 nm. The methodology of *b_b_*(*λ_i_*) measurements, calibration procedures, and subsequent determination of *b_b_*(*λ_i_*) have been described by Maffione and Dana [61]. To estimate *b_b_*(*λ_i_*) [61,62] we used values of volume scattering function $\left(\beta \left(\Psi \right)\right)$ at an angle of 140^o^. We took data from absorption and attenuation meter ac-9 (WET Labs, Philomath, OR, USA) to a procedure called sigma correction (a correction for an incomplete recovery of backscattered light in highly attenuating waters, according to the User’s Manual [63]).

The *R_rs_*(*λ_i_*) was also calculated using data obtained from radiometer OCP-100 (just below the water). The *R_rs_*(*λ_i_*) values estimated by OCP-100 were used to calculate the *f/Q* ratio. OCP-100 measured upwelling radiance (*L_u_*(*λ_i_*)) and downwelling irradiance (*E_d_*(*λ_i_*)) on seven channels: *λ_i_* = 412, 443, 490, 510, 555, 670 and 683 nm. We used nearest-neighbour interpolation to estimate wavelengths *L_u_*(*420*), *E_d_*(*420*), *L_u_*(488), *E_d_*(488), *L_u_*(620) and *E_d_*(620), between the measured data wavelengths. The *L_u_*(*λ_i_*) values obtained from the OCP-100 m were corrected for self-shading effects [64,65]. The values of *R_rs_*(*λ_i_*) were calculated using the following equation:

(4) $$R_{rs}=\frac{L_{u}\left(0^{-}\right)}{E_{d}\left(0^{-}\right)},$$

where (0^−^) means just below the water.

Previous studies have shown that *R_r_*(*λ_i_*)*_s_* is also proportional to the ratio of *b_b_* and *a* based on Morel and Gentili [66]. In our study we used the following equation [66]:

(5) $$R_{rs}\left(\lambda_{i}\right)={\left[\frac{f}{Q}\right]}_{\lambda_{i}}\;\frac{b_{b}\left(\lambda_{i}\right)}{a(\lambda_{i})+b_{b}\left(\lambda_{i}\right)},$$

where:

(6) $$Q=\frac{E_{u}\left(0^{-}\right)}{L_{u}\left(0^{-}\right)},$$

and (*E_u_*(0^–^)) is upwelling irradiance.

The *Q* factor is a measure of the non-isotropy of the upward radiance field [67]. The *f* factor depends on the wavelength of the light, the solar zenith angle, and the single scattering albedo. The variability of the *f/Q* ratio and its impact on the *R_rs_* values has been the subject of many theoretical analyses [68]. In 2002, Morel et al. [69] established that for Case 2 water, the *f/Q* ratio ranges from 0.07 to 0.18 sr^−1^, and it depends on the concentration of *Chl a* and the illumination of the area (e.g., solar zenith angle, waves, etc.). These theoretical analyses were supported by empirical research conducted by Voss and Morel in 2005 [70]. However, for Case 2 waters, determining the *f/Q* ratio is not easy and requires a strictly local approach. In 2003, D’Sa and Miller [35] determined that at the mouth of the Mississippi River in the Gulf of Mexico, this ratio ranges from 0.09 to 0.12 sr^−1^. We attempted to determine the empirical *f/Q* values for the analysed areas and established that the *f/Q* ratio for the southern Baltic water ranges from 0.07 to 0.13 sr^−1^ and depends on the wavelength (it increases with the wavelength). For this purpose, we used measurements of *L_u_*(*λ_i_*), *E_d_*(*λ_i_*), *b_b_*(*λ_i_*) and *a*(*λ_i_*) for four wavelengths of light (*λ_i_* = 420, 488, 555 and 620 nm). Next, using Equations (4) and (5), we calculated *f/Q* values for the four tested wavelengths.

## 3. Results

### 3.1. Analysis of the Impact of Biogeochemical Components on the Optical Properties of the Southern Baltic Coastal Waters

**Figure 3:**
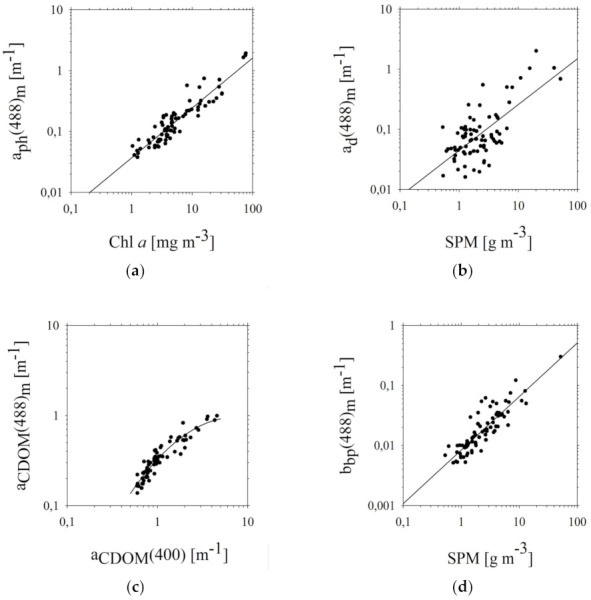
Relationships between (**a**) *a_ph_* and *Chl a*, (**b**) *a_d_* and *SPM*, (**c**) *a_CDOM_* and *a_CDOM_*(400), (**d**) *b_bp_* and *SPM* (for a wavelength of 488 nm) in the selected areas of the southern Baltic [line approximation by equations (**a**) 8, (**b**) 12, (**c**) 20, and (**d**) 16].

We checked many correlations between optical properties and biogeochemical components of the southern Baltic waters. First, the relations between *IOPs* ((*a_ph_*(*λ_i_*), *a_d_*(*λ_i_*), *a_CDOM_*(*λ_i_*) and *b_bp_*(*λ_i_*)) and concentration of a single parameter (*Chl a*, *SPM and a_CDOM_*(400)) were statistically approximated for all four wavelengths. Figure 3 shows the relationships between the empirically determined *a_ph_*(*λ_i_*), *a_d_*(*λ_i_*), *a_CDOM_*(*λ_i_*) (Figure 3a–c) and *b_bp_*(*λ_i_*) (Figure 3d) for a wavelength 488 nm and concentration of biogeochemical components of water (*Chl a*, *SPM* and *a_CDOM_*).

We observed a positive correlation between *a_ph_*(488), *a_d_*(488), *a_CDOM_*(488), and *b_bp_*(488) and the concentration of biogeochemical components. In Figure 3a, we see that correlation between *a_ph_*(488) and *Chl a* is better than the relationships: *a_d_*(488)*-SPM*, *a_CDOM_*(488)*-a_CDOM_*(400), and *b_bp_*(488)*-SPM.* This relationship is also the most linear in contrast to the correlation between *a_CDOM_*(488) and *a_CDOM_*(400), which was the nature of absorption by CDOM is particularly complex due to the variety of chemical structures of dissolved substances in natural waters and the differentiation of the interaction of their molecules with electromagnetic radiation. They include both saturated substances, which practically do not absorb light in the visible range, and unsaturated substances, which strongly absorb light. Therefore, in the *a_CDOM_* analyses, we adopted *a_CDOM_* (400) commonly used for many years also by other authors [4,37,71], namely the *a_CDOM_* for the selected reference wavelength, *a_CDOM_*(*λ_ref_*). We assumed that λ_ref_ = 400 nm.

**Figure 4:**
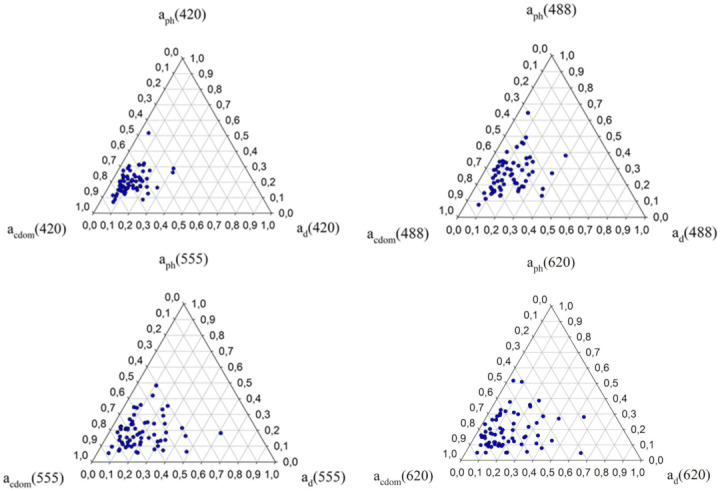
Ternary plots of the relative contribution of CDOM (*a*_CDOM_), non-phytoplankton pigments (*a_d_*) and phytoplankton pigments (*a_ph_*) to total absorption by non-water constituents at four wavelengths in the selected areas of the southern Baltic. The relative contribution of a given component was calculated as the ratio of the absorption coefficient of that component (e.g., *a*_ph_(*λ_i_*)) and the sum of the absorption coefficients of all three components [*a*_CDOM_(*λ_i_*) + *a_d_*(*λ_i_*) + *a_ph_*(*λ_i_*)].

Figure 4 shows the absorption budget for *a_d_*(*λ_i_*), *a_ph_*(*λ_i_*), and *a_CDOM_*(*λ_i_*) in the chosen areas of the southern Baltic. We can see that *a*_CDOM_ has the greatest contribution in the total absorption at a wavelength of 420 nm and its average percentage is 68%, while the average shares of other absorption coefficients are *a_ph_*-20% and *a_d_*-12%.

The relationships between *a_ph_*(*λ_i_*) and *Chl a* (Equations (7)–(10)) and also between *a_d_*(*λ_i_*) and *SPM* (Equations (11)–(14)), or between *b_bp_*(*λ_i_*) and *SPM* (Equations (15)–(18)) are well approximated by power functions such as:

*a_ph_*(420)*_cal_* = 0.056(*Chl a*)^0.827^, (7)

*a_ph_*(488)*_cal_* = 0.037(*Chl a*)^0.820^, (8)

*a_ph_*(555)*_cal_* = 0.013(*Chl a*)^0.815^, (9)

*a_ph_*(620)*_cal_* = 0.008(*Chl a*)^0.926^, (10)

*a_d_*(420)*_cal_* = 0.071(*SPM*)^0.809^, (11)

*a_d_*(488)*_cal_* = 0.045(*SPM*)^0.762^, (12)

*a_d_*(555)*_cal_* = 0.031(*SPM*)^0.646^, (13)

*a_d_*(620)*_cal_* = 0.002(*SPM*)^0.592^, (14)

*b_bp_*(420)*_cal_* = 0.011(*SPM*)^0.911^, (15)

*b_bp_*(488)*_cal_* = 0.008(*SPM*)^0.891^, (16)

*b_bp_*(555)*_cal_* = 0.007(*SPM*)^0.935^, (17)

*b_bp_*(620)*_ca_*_l_ = 0.005(*SPM*)^0.881^. (18)

Relationships between *a_CDOM_*(*λ_i_*) and *a_CDOM_*(400) (Equations (19)–(22)) are well approximated by second order non-linear exponential functions:

(19) $$a_{CDOM}{\left(420\right)}_{cal}=10^{[-0.077+\left(a_{CDOM}\left(400\right){)}^{2}+1.006\;a_{CDOM}\left(400\right)-0.132\right]},$$

(20) $$a_{CDOM}{\left(488\right)}_{cal}=10^{[-0.624+\left(a_{CDOM}\left(400\right){)}^{2}+1.077\;a_{CDOM}\left(400\right)-0.485\right]}$$

(21) $$a_{CDOM}{\left(555\right)}_{cal}=10^{[-1.037+\left(a_{CDOM}\left(400\right){)}^{2}+1.072\left(400\right)-0.689\right]}$$

(22) $$a_{CDOM}{\left(620\right)}_{cal}=10^{[-1.488+\left(a_{CDOM}\left(400\right){)}^{2}+1.136\left(400\right)-0.794\right]}$$

**Figure 5:**
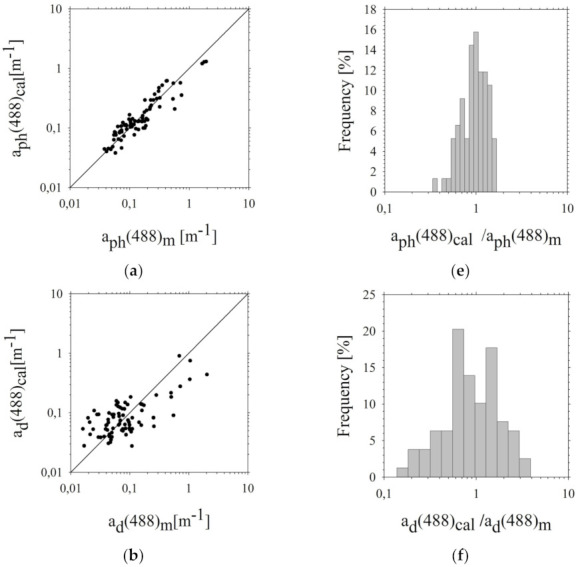

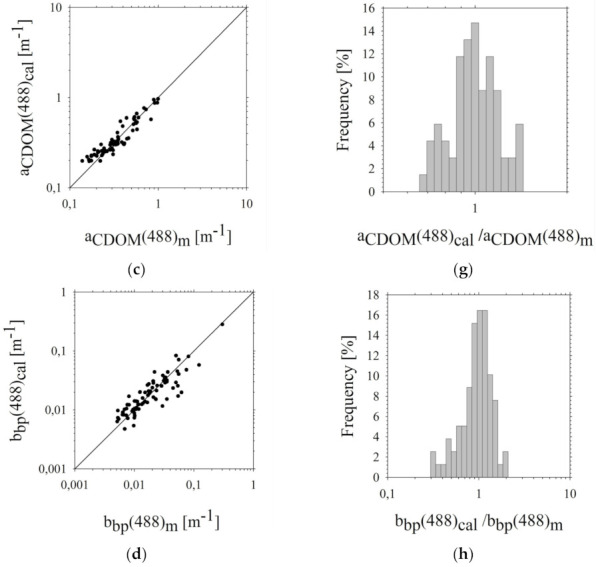
Comparison of: *a_ph_*(*λ_i_*), *a_d_*(*λ_i_*), *a_CDOM_*(*λ_i_*) and *b_bp_*(*λ_i_*) measured (*X*(*λ_i_*)*_m_*) and calculated (*X*(*λ_i_*)*_cal_*) (**a**–**d**) using algorithms: (**a**) Equation (8), (**b**) Equation (12), (**c**) Equation (20) and (**d**) Equation (16) for a single wavelength (488 nm)-in the chosen areas of the southern Baltic. The solid line represents the function (*X*(*λ_i_*)*_m_* = *X*(*λ_i_*)*_cal_*). The probability density distribution of the ratio of calculated (*X*(*λ_i_*)*_cal_*) to measured (*X*(*λ_i_*)*_m_*) light absorption and backscattering coefficients (**e**–**h**) for wavelength 488 nm.

The accuracy of the approximating functions presented above was assessed by comparing the empirical *a_d_*(*λ_i_*), *a_ph_*(*λ_i_*), and *a_CDOM_*(*λ_i_*) and *b_bp_*(*λ_i_*) coefficients with the values of the coefficients calculated based on Equations (7)–(22) (Figure 5). In addition, statistical errors were determined (Table 1).

The following arithmetic and logarithmic statistical metrics were used to assess the uncertainty of the empirical relationships and models (X_i,m_—measured values; X_i,cal_—estimated values [the subscript m stands for “measured”; cal—stands for “calculated”]):

1. relative mean error (systematic error):

(23) $$\langle \varepsilon \rangle =N^{-1}\sum_{i}\;\;\;\varepsilon_{i\;}(where\;\varepsilon_{i}=\left(\frac{X_{i,cal}-X_{i,\;m}}{X_{i,\;m}}\right),$$

2. standard deviation (statistical error) of ε (RMSE-root mean square error):

(24) $$\sigma_{\varepsilon }=\sqrt{\frac{1}{N}\left(\sum_{}^{}{\left(\varepsilon_{i}-\langle \varepsilon \rangle \right)}^{2}\right)},$$

3. mean logarithmic error:

(25) $${\langle \varepsilon \rangle }_{g}=10^{\left[\langle log\left(\frac{X_{i,cal}}{X_{i,\;m}}\right)\rangle \right]}-1,$$

4. standard error factor:

(26) $$x=10^{\sigma_{log}},$$

5. statistical logarithmic errors:

(27) $$\sigma_{+}=x-1\;\;\;\;\sigma_{-}=\frac{1}{x}-1,$$

6.
$\sigma_{log}-standard\;deviation\;of\;the\;set\;log\left(\frac{X_{i,cal}}{X_{i,\;m}}\right);$

7.
$\langle log\left(\frac{X_{i,cal}}{X_{i,\;m}}\right)\rangle -mean\;of\;log\left(\frac{X_{i,cal}}{X_{i,\;m}}\right).\;$

The relatively small error values of *a_p_*_h_ quoted in Table 1 indicate fairly accurate selection of the approximating functions. Similar functions approximating the relationship *a_ph_*(*λ_i_*) = *f*(*Chl a*) were used for Case 1 waters by Bricaud et al. [5], and for the waters of the Baltic Sea by Woźniak, Meler et al. [2,31,32,72].

In the case of *a_d_*(*λ_i_*), the error values amount to several dozen or even exceed one hundred per cent. This can be explained partly by methodological reasons. Measurements of *a_d_*(*λ_i_*) and *SPM* are very complex and subject to significant errors. There is a large variety of *SPM* in Case 2 waters, which translates into different density and absorption capacities. This is undoubtedly a significant cause of high errors in estimating these coefficients based solely on the total weight of the suspended matter. The non-algal particles consist of organic detritus and mineral particles. Therefore, further analyses took into account the types of suspended particles, i.e., their organic and inorganic fractions.

In the case of the *a_CDOM_*(*λ_i_*), the approximation errors are relatively low and amount to a few per cent for the coefficients relating to the wavelength of 420 nm, i.e., the closest reference wave (Table 1). Moving further from this wavelength, the errors of the estimated *a_CDOM_*(*λ_i_*) increase, reaching several per cent for *λ_i_* = 488 nm, about 30% for *λ_i_* = 555 nm, and nearly 40% for *λ_i_* = 620 nm. The errors in the one-parameter estimation for *a_CDOM_*(*λ_i_*) presented above indicate that its accuracy is satisfactory. Therefore, we found that the determined model descriptions of *a_CDOM_*(*λ_i_*) (Equations (19)–(22)) can be used successfully in the model of the reflectance coefficient for selected areas of the southern Baltic.

The accuracy of *b_bp_*(*λ_i_*) determination based on the *SPM* obtained using Equations (15)–(18) (Table 1) is definitely better than the accuracy of determining *a_d_*(*λ_i_*) based on the analogous dependence of these coefficients on the *SPM*. In the case of absorption, these errors are usually twice as great as in the case of scattering. Given that in the natural environment the values of *b_bp_*(*λ_i_*) as well as *a_ph_*(*λ_i_*) vary over several orders of magnitude, error values mostly in the range from about 30% to about 50% for *b_b_*(*λ_i_* of the suspended particles are acceptable. However, we have attempted to improve the accuracy of the estimated *b_bp_*(*λ_i_*) and *a_ph_*(*λ_i_*) values by including additional biogeochemical parameters: *SPM_inorg_* for *b_bp_*(*λ_i_*) and ∑*C* for *a_ph_*(*λ_i_*) (as in the case of *a_d_*(*λ_i_*)).

**Figure 6:**
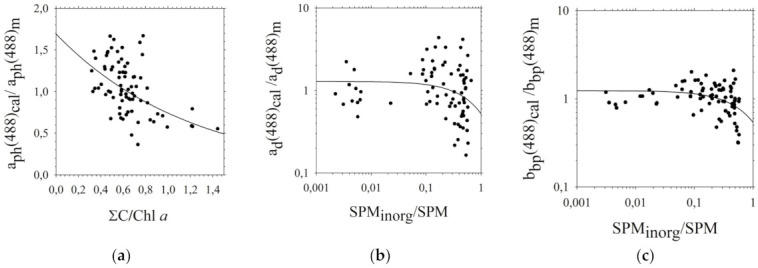
Comparison of: (**a**) *a_ph_* calculated using Equation (8) and the ratio of *Chl a* and ∑*C*, (**b**) *a_d_* calculated using Equation (12) and the ratio of *SPM* and *SPM_inorg_*, (**c**) *b_bp_* calculated using Equation (16) and the ratio of *SPM* and *SPM_inorg_*, (for wavelength of 488 nm) in the selected areas of the southern Baltic [the line approximation by equations (**a**) 27 (**b**) 28 and (**c**) 29] The coefficient of determination (*R^2^*): (**a**) 0.27, (**b**) 0.02, (**c**) 0.04.

Case 2 waters are chemically more complex than Case 1 waters. Therefore, interpretation of optical signals coming from such water areas is quite difficult. For this reason, the relationships determined in the first stage were subjected to subsequent statistical analyses, during which additional parameters were introduced: in the case of *a_ph_*-∑*C*, and in the case of *a_d_* and *b_bp_*-*SPM_inorg_* (Figure 6).

The ratio of calculated: *a_ph_*(*λ_i_*), *a_d_*(*λ_i_*) and *b_bp_*(*λ_i_*) values determined by Equations (8), (12) and (16), to the measured values of *a_ph_*(*λ_i_*), *a_d_*(*λ_i_*) and *b_bp_*(*λ_i_*) was compared with the ratio of of *Chl a* and ∑*C* (for *a_ph_*(*λ_i_*)) and the ratio of *SPM* and *SPM_inorg_* (for *a_d_*(*λ_i_*) and *b_bp_*(*λ_i_*)) (Figure 6, Equations (27)–(29)).

(28) $$\frac{a_{ph}{\left(488\right)}_{cal}}{a_{ph}{\left(488\right)}_{m}}=1.692e{\;}^{-0.824\frac{\Sigma C}{Chl\;a}}{}^{}\;,$$

(29) $$\frac{a_{d}{\left(488\right)}_{cal}}{a_{d}{\left(488\right)}_{m}}=e{\;}^{-0.903\frac{SPM_{inorg}}{SPM}}{}^{},$$

(30) $$\frac{b_{bp}{\left(488\right)}_{cal}}{b_{bp}{\left(488\right)}_{m}}=e{\;}^{-0.827\frac{SPM_{inorg}}{SPM}}{}^{}.$$

Among the various analysed forms of approximating functions, exponential functions turned out to be the most appropriate (Equations (30)–(41)).

(31) $$a_{ph}{\left(420\right)}_{cal}=0.041{\left(Chl\;a\right)}^{0.827}e{\;}^{0.493\frac{\Sigma C}{Chl\;a}}{}^{},$$

(32) $$a_{ph}{\left(488\right)}_{cal}=0.022{\left(Chl\;a\right)}^{0.820}e{\;}^{0.824\frac{\Sigma C}{Chl\;a}}{}^{},$$

(33) $$a_{ph}{\left(555\right)}_{cal}=0.011{\left(Chl\;a\right)}^{0.815}e{\;}^{0.257\frac{\Sigma C}{Chl\;a}}{}^{},$$

(34) $$a_{ph}{\left(620\right)}_{cal}=0.007{\left(Chl\;a\right)}^{0.926}e{\;}^{0.261\frac{\Sigma C}{Chl\;a}}{}^{},$$

(35) $$a_{d}{\left(420\right)}_{cal}=0.057{\left(SPM\right)}^{0.809}e{\;}^{0.750\frac{SPM_{inorg}}{SPM}}{}^{},$$

(36) $$a_{d}{\left(488\right)}_{cal}=0.035{\left(SPM\right)}^{0.762}e{\;}^{0.903\frac{SPM_{inorg}}{SPM}}{}^{},$$

(37) $$a_{d}{\left(555\right)}_{cal}=0.022{\left(SPM\right)}^{0.646}e{\;}^{1.157\frac{SPM_{inorg}}{SPM}}{}^{},$$

(38) $$a_{d}{\left(620\right)}_{cal}=0.015{\left(SPM\right)}^{0.592}e{\;}^{1.542\frac{SPM_{inorg}}{SPM}}{}^{},$$

(39) $$b_{bp}{\left(420\right)}_{cal}=0.009{\left(SPM\right)}^{0.911}e{\;}^{0.337\frac{SPM_{inorg}}{SPM}}{}^{},$$

(40) $$b_{bp}{\left(488\right)}_{cal}=0.006{\left(SPM\right)}^{0.891}e{\;}^{0.827\frac{SPM_{inorg}}{SPM}}{}^{},$$

(41) $$b_{bp}{\left(555\right)}_{cal}=0.005{\left(SPM\right)}^{0.935}e{\;}^{0.977\frac{SPM_{inorg}}{SPM}}{}^{},$$

(42) $$b_{bp}{\left(620\right)}_{cal}=0.004{\left(SPM\right)}^{0.881}e{\;}^{1.230\frac{SPM_{inorg}}{SPM}}{}^{}.$$

**Figure 7:**
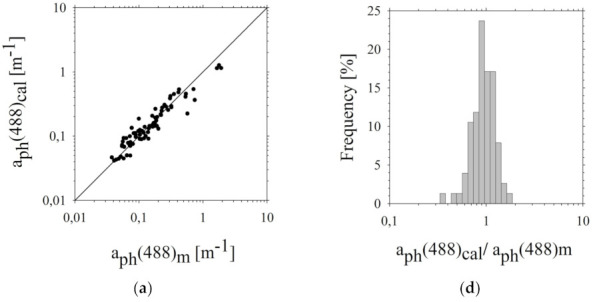

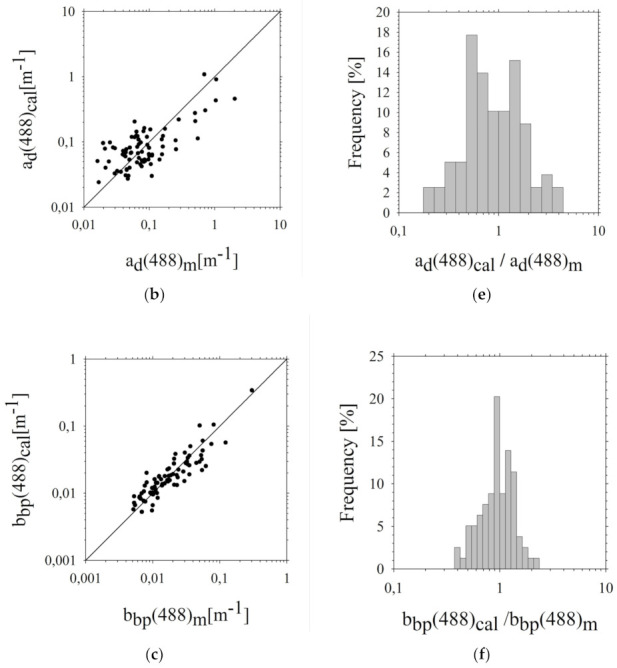
Comparison of: *a_ph_*(*λ_i_*), *a_d_*(*λ_i_*), and *b_bp_*(*λ_i_*) measured (*X*(*λ_i_*)*_m_*) and calculated (*X*(*λ_i_*)*_cal_*) (**a**–**c**) using algorithms: (**a**) Equation (32), (**b**) Equation (36), (**c**) Equation (40) for a single wavelength (488 nm) in the chosen areas of the southern Baltic. The solid line represents the function (*X*(*λ_i_*)*_m_* = *X*(*λ_i_*)*_cal_*). The probability density distribution of the ratio of calculated light absorption and backscattering coefficients (*X*(*λ_i_*)*_cal_*) to measured (*X*(*λ_i_*)*_m_*) (**d**–**f**) for a single wavelength (488 nm). The coefficient of determination (*R^2^*): (**a**) 0.92, (**b**) 0.49, (**c**) 0.87.

Graphical comparison of the measured empirical values *a_ph_*(488)*_m_*, *a_d_*(488)*_m_* and *b_bp_*(488)*_m_* with their respective values estimated using the two-parameter model of *a_ph_*(488)*_cal_*, *a_d_*(488)*_cal_* and *b_bp_*(488)*_cal_* and ratio distribution histograms (*a_ph_*(488)*_cal_/a_ph_*(488)*_m_*) is presented in Figure 7. Statistical errors (according to linear and logarithmic statistics) of these estimates were also determined (Table 2).

The values of respective errors presented in Table 2 indicate that the dependencies of *a_ph_*(*λ_i_*) on ∑*C* and *Chl a* are more accurate than the dependence of *a_ph_*(*λ_i_*) on the concentration of only one parameter (see Table 1, Equations (31)–(34)). The differences between the errors determined for both obtained relationships are in the range from 0.2% to approximately 6.5%. They are respectively equal for the successive wavelengths: (1) for arithmetic statistics −2.1%, 3.8%, 1.6% and 0.2% and (2) for logarithmic statistics −2.5%, 6.4%, 1.1% and 0.6%. Thus, the model described by Equations (31)–(34), taking into account two parameters (∑*C* and *Chl a*), is more suitable for the determination of *a_ph_*(*λ_i_*).

As can be seen from the verification results for the *a_d_* (Table 2, Equations (35)–(38)), systematic errors are relatively small compared to statistical errors. The standard error factor determined for the model of *a_d_*(*λ_i_*) is characterised by a similar tendency as the model error factor *a_ph_*(*λ_i_*) (Table 1, Equations (31)–(34)), i.e., its value increases with increasing wavelength. For *λ_i_* = 420 nm, it is x = 1.91, while for *λ_i_* = 620 nm, it is higher by almost a half and amounts to x = 2.67. As in the case of *a_ph_*(*λ_i_*), we observe an improvement in the *a_d_*(*λ_i_*) estimations after introducing an additional parameter. Thus, also in this model, the dependency of *a_d_*(*λ_i_*) on the *SPM* and *SPM_inorg_* is better than the dependence of these coefficients on the concentration of only one parameter, *SPM*.

The magnitude of *b_bp_*(*λ_i_*) statistical error values (estimated based on *SPM* and *SPM_inorg_*—Equations (39)–(42)) ranges from 30% to 50% (Table 2) and is slightly smaller than the error magnitudes of *b_bp_*(*λ_i_*) estimated based on *SPM*—Equations (15)–(18) (Table 1). The differences between these error magnitudes range from about 0% to 9% depending on the wavelength. Additionally, they are also different in the case of arithmetic and logarithmic statistics. In the arithmetic statistics they are close to 0% (for the wavelengths 420, 488, and 555 nm) or approximately 2% (for the light with a wavelength of 620 nm), while logarithmic statistics they are approximately 0.7% for 420 nm, 4.3% for 488 nm, 6.2% for 555 nm, and 8.9% for 620 nm. This means that the variation in the chemical nature of the suspended particles affects their optical scattering capacity only slightly, just as it happened in the case of the *a_d_* albeit to a lesser extent.

### 3.2. The Five-Parameter Semi-Empirical Model of R_rs_(*λ_i_*) of the Southern Baltic Coastal Waters

The culmination of the above analyses is a model representation of *R_rs_*(*λ_i_*) in selected sea areas of the southern Baltic, which allows us to calculate *R_rs_*(*λ_i_*) for four wavelengths in the visible light range. For this purpose, we used Equation (5) and we based on the known spectra of *a*(*λ_i_*) and *b_b_*(*λ_i_*), or known concentrations of biogeochemical constituents occurring in these waters (Equations (43)–(46), Table 3).

(43) $$b_{bp}{\left(\lambda_{i}\right)}_{cal}=C(\lambda_{i}){\left(SPM\right)}^{B(\lambda_{i})}\;e^{D(\lambda_{i})\frac{SPM_{inorg}}{SPM}\;}{}_{},$$

(44) $$a_{ph}{\left(\lambda_{i}\right)}_{cal}=G(\lambda_{i}){\left(Chl\;a\right)}^{F(\lambda_{i})}e^{H(\lambda_{i})\frac{\Sigma C}{Chl\;a}\;},$$

(45) $$a_{d}{\left(\lambda_{i}\right)}_{cal}=K(\lambda_{i}){\left(SPM\right)}^{J(\lambda_{i})}e^{L(\lambda_{i})\frac{SPM_{inorg}}{SPM}\;}{}_{},$$

(46) $$a_{CDOM}{\left(\lambda_{i}\right)}_{cal}=10^{\left[-M(\lambda_{i}\right){\left(a_{CDOM}\left(400\right)\right)}^{2}+N(\lambda_{i})a_{CDOM}\left(400\right)-P{\left(\lambda_{i}\right)}^{}]}{}_{}.$$

We established that the values of *f/Q* for the southern Baltic water are: 0.07 for 420 nm, 0.10 for 488, 0.12 for 555 nm and 0.13 for 555 nm (Table 3).

### 3.3. Assessment of Estimation Errors of the Five-Parameter R_rs_(*λ_i_*) Model

**Figure 8:**
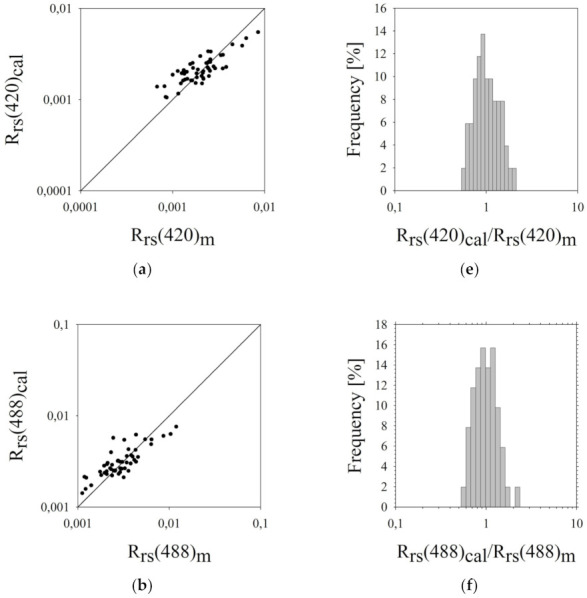

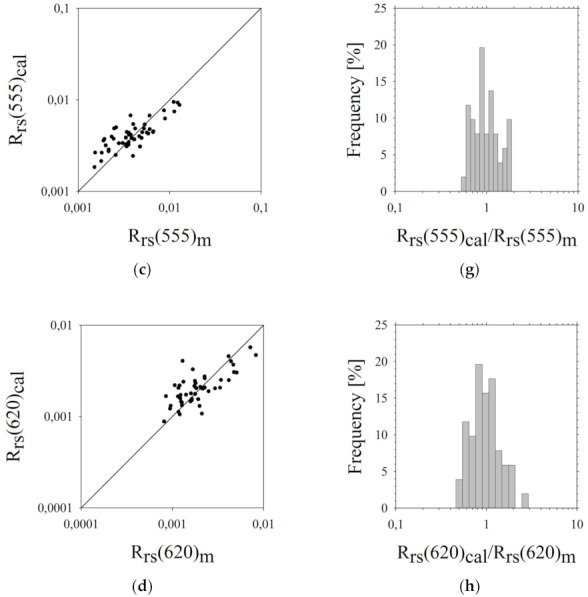
(**a**–**d**) Comparison of the measured *R_rs_*(*λ_i_*)*_m_* and calculated *R_rs_*(*λ_i_*)*_cal_* (Equations (43)–(46), Table 3) for four wavelengths (*λ_i_* = 420 nm, 488 nm, 555 nm and 620 nm) in the selected areas of the southern Baltic. The solid line represents the function (*R_rs_*(*λ_i_*)*_m_* = *R_rs_*(*λ_i_*)*_cal_*) (**e**–**h**). The probability density distribution of the ratio of calculated *R_rs_*(*λ_i_*)*_ca_* to measured *R_rs_*(*λ_i_*)*_m_*.

The estimation errors are presented in Table 4. The probability density distribution of the ratio *R_rs_*(*λ_i_*)*_cal_/R_rs_*(*λ_i_*)*_m_* is shown in Figure 8. Moreover, the figure presents a graphical comparison of the empirical values of *R_rs_*(*λ_i_*)*_m_* with values calculated using the *R_rs_*(*λ_i_*)*_cal_* model.

In the case of the five-parameter *R_rs_* model, the dispersion of points is significant (especially for the wavelength of 620 nm) and in all graphs, it takes a very similar form. The greater scatter of points for the 620 nm wavelength can be explained by the fact that this region of the spectrum is especially influenced by the processes of light absorption by *SPM* (both organic and non-organic particles). The water areas in which the research was conducted are centres very rich in both dissolved and suspended matter.

The error magnitudes of the *R_rs_*(*λ_i_*) values calculated in the five-parameter model do not exceed 50% for all wavelengths (Table 4). The result is satisfactory since the coastal zone of the southern Baltic is the area characterized by high water dynamics and values of physicochemical parameters can be high, especially in the areas of river mouths. Thus, in such conditions it is extremely difficult to find bio-optical algorithms that would enable accurate estimation of optical quantities based on e.g., the concentration of various types of optically active dopant.

## 4. Discussion

### 4.1. Reference to the Main Research Objectives

The primary goal of our work was to find the local, multiparameter algorithms between *IOPs* (*a_ph_*(*λ_i_*), *a_d_*(*λ_i_*), *a_CDOM_*(*λ_i_*), *b_bp_*(*λ_i_*)) and *AOPs* (*R_rs_*(*λ_i_*)) and the concentrations of biochemical components of water (*Chl a*, ∑*C*, *SPM*, *SPM_inorg_*, *a_CDOM_*(400)) in selected Case 2 waters.

Achieving the assumed objectives required a wide range of experimental work, both in situ (measurements of *b_bp_*(*λ_i_*) and in vitro (*a_ph_*(*λ_i_*), *a_d_*(*λ_i_*), *a_CDOM_*(*λ_i_*), *a_CDOM_*(400), *Chl a*, ∑*C*, *SPM*, *SPM_inorg_*). It was also necessary to carry out a theoretical analysis of *a*(*λ_i_*) and *b_b_*(*λ_i_*) and to establish the relationship between these optical properties and biogeochemical parameters of seawater components.

### 4.2. Summary of the Main Findings of the Article

The presented results meet all the aims assumed and constitute a significant contribution to the knowledge on the optics of Case 2 waters represented by the coastal regions of the southern Baltic. Based on statistical analyses of empirical material, mathematical equations describing the relationships between the coefficients of light absorption by the main groups of absorbents in the studied areas and *b_b_*(*λ_i_*) by suspended matter and the concentrations of these components in the water were established. Moreover, model descriptions of *a*(*λ_i_*) and *b_b_*(*λ_i_*) for selected waters of the southern Baltic were developed, allowing us to calculate *a*(*λ_i_*) and *b_b_*(*λ_i_*) for the four selected wavelengths, based on the known concentrations of these components in the waters. Finally, as a result of the analyses conducted, a set of mathematical equations for calculating *R_rs_*(*λ_i_*) spectra in the visible light range (for wavelengths *λ_i_* = 420, 488, 555, and 620 nm) was developed. The formulas are based on the known spectra of *a*(*λ_i_*) and *b_b_*(*λ_i_*) in the coastal waters of the southern Baltic or based on the knowledge of the concentration of admixture components present there.

In 2014, Woźniak [27] presented simple statistical formulas for estimating various biogeochemical properties of *SPM* in the southern Baltic. He used empirical data and found statistical formulas for estimating biogeochemical properties of *SPM* based on *IOPs*. He showed that empirical formulas, although encumbered by statistical errors, can be used to estimate the biogeochemical properties of SPM for the region of the southern Baltic, thus for the derivation of local remote-sensing algorithms. In 2020, Woźniak and Meler [2] made similar attempts to create a simple, local *R_rs_* (*λ_i_*) model from a nearshore location in the Baltic Sea. Their algorithms were based on empirical data of *IOPs.* They used the Hydrolight model to calculate *R_rs_*.

### 4.3. Limitations of Our Research

In Case 2 waters, the values of *IOPs* and *AOPs* depend on the season and geographic location. Certain “deviations from the norm” may also be observed, which is common for Case 2 waters, because in these areas, seasonal cycles are often modified by various types of irregular anomalies caused by e.g., weather factors. Therefore, the measurements of biogeochemical parameters and optical properties of these waters must be carried out simultaneously, regularly, and often. Data collected over a long period allow us to capture the seasonal variability of these quantities and develop remote monitoring algorithms.

### 4.4. Recommendations for Future Research

To improve the accuracy of estimating concentrations of optically active components in Case 2 waters using remote-sensing methods, it is necessary, first of all, to understand the bio-optical properties of these components. Seawater components such as phytoplankton, dissolved and suspended organic and inorganic matter determine *IOPs*, and these, in turn, affect *AOPs*. Because within Case 2 waters, the values of *IOPs* and *AOPs* may vary within several orders of magnitude, it is reasonable to establish individual and seasonal links between optical properties and the concentration of individual components in the water for each of these water areas.

Therefore, it is justified to conduct the widest possible research on the physical, optical, and chemical properties of the Case 2 waters to construct local algorithms for them. In the future, it is worth supplementing the empirical data banks obtained from these water areas with new data. This will enable a better estimation of the size and speed of changes in the relationship between *IOPs* and *AOPs* and the concentration of *SPM* in Case 2 waters.

## 5. Conclusions

As a result of the analysis of dependence of *a_ph_*(*λ_i_*) on the concentrations of *Chl a*, it was found that *a_ph_*(*λ_i_*) increases with a rise in the values of *Chl a* concentrations. However, these values are not directly proportional to each other, as the increase in *a_ph_*(*λ_i_*) is lower than the increase in *Chl a* concentrations. These regularities are described well by the power function (Equations (7)–(10)).

Statistical analyses showed that the estimation of *a_ph_*(*λ_i_*) in chosen areas of the southern Baltic using model descriptions of these coefficients as a function of two independent variables (see Equations (31)–(34)), i.e., *Chl a* and ∑*C*, is better than estimates of *a_ph_*(*λ_i_*) as a function of *Chl a*. The calculated differences between the estimation errors for both of these model descriptions are approximately 2.5%, ranging from about 1% to about 5%, and they depend on the wavelength (see Table 1 and Table 2).

The analysis of the dependence of *a_d_*(*λ_i_*) values on the total concentrations of *SPM* in seawater indicated that these coefficients rise with an increase in suspended matter concentration, although the rise is not linear. The increase in *a_d_*(*λ_i_*) is less intense than the increase in the total suspended matter concentration. These regularities are described by hyperbolic functions (see Equations (11)–(14)). The statistical analyses performed showed that the accuracy of the estimation of *a_d_*(*λ_i_*) increases when they are described as functions of two independent variables (i.e., *SPM* and *SPM_inorg_* of the suspended matter fraction). These tendencies are described well by hyperbolic functions (see Equations (35)–(38)). The calculated differences between the estimation errors for both of these model descriptions ranging from about small negative values (i.e., when the accuracy of the estimation based on a function with one independent variable is better than the accuracy of using the dependence on two variables) to about 12.4% and they also depend on the wavelength (see Table 1 and Table 2).

The analyses showed that *a_CDOM_*(*λ_i_*) in seawater rise with an increase in the value of the optical index of their concentrations, *a_CDOM_*(400). They can be described with satisfactory accuracy as a hyperbolic function of a single variable (see Equations (19)–(22)). In the case of *a_CDOM_*, the approximation errors are relatively low for short waves (a few percent for *λ* = 420 nm) and they increase with the wavelength approaching 40% for *λ* = 620 nm (see Table 1).

Just as the absorption capacity, the backscattering properties of seawater also depend on *SPM* and *SPM_inorg_*. These relationships can be presented in the form of hyperbolic expressions (see Equations (39)–(42)). The statistical errors in the case of *b_bp_* as a function of two independent variables range from about 30% to about 50%, and they depend on the wavelength (see Table 1 and Table 2). Their magnitude is lower than magnitude of statistical errors in the case of *b_bp_* as a function of one independent variable.

The analyses showed that *R_rs_*(*λ_i_*) in the waters of the southern Baltic can be described with satisfactory accuracy using the five-parameter model presented in this paper, the parameters in which are *Chl a*, ∑*C*, *SPM*, *SPM_inorg_*, and *a_CDOM_*(400). In this case, the statistical errors do not exceed 50% for all wavelengths, about 35% for *λ* = 420 nm and 488 nm, about 38% for *λ* = 555 nm and about 38% for *λ* = 420 nm (see Table 4).

## Figures and Tables

**Table 1:** The relative errors in the approximations.

Step 1. 1-Parameter Model of *IOPs*	Arithmetic Statistic		Logarithmic Statistic			
	Systematic Error	Statistical Error	Systematic Error	Standard Error Factor	Statistical Error	
	<ε> [%]	σ_ε_ [%]	<ε>_g_ [%]	x	σ_ε+_ [%]	σ_ε−_ [%]
*a_ph_*(420) Equation (7)	4.39	30.97	−1.54 × 10 ^−2^	1.35	34.66	−25.74
*a_ph_*(488) Equation (8)	4.91	31.10	7.1 × 10 ^−3^	1.38	37.97	−27.52
*a_ph_*(555) Equation (9)	4.46	30.80	3.3 × 10 ^−3^	1.35	35.01	−25.93
*a_ph_*(620) Equation (10)	5.46	34.01	1.1 × 10 ^−3^	1.40	39.90	−28.52
*a_d_*(420) Equation (11)	23.7	86.1	−1.4 × 10 ^−3^	1.94	94.3	−48.5
*a_d_*(488) Equation (12)	26.4	89.7	−5.9 × 10 ^−3^	2.03	103.2	−50.8
*a_d_*(555) Equation (13)	38.1	115.9	2.7 × 10 ^−3^	2.30	130.4	−56.6
*a_d_*(620) Equation (14)	65.5	188.9	9.1 × 10 ^−3^	2.79	179.1	−64.2
*a_CDOM_*(420) Equation (19)	0.23	7.08	−0.01	1.07	7.25	−6.76
*a_CDOM_*(488) Equation (20)	1.69	18.60	0.01	1.20	20.31	−16.88
*a_CDOM_*(555) Equation (21)	4.71	32.91	0.01	1.35	35.37	−26.13
*a_CDOM_*(620) Equation (22)	6.94	40.23	0.01	1.45	44.52	−30.81
*b_bp_*(420) Equation (15)	8.87	44.52	8.25 × 10 ^−3^	1.53	52.93	−34.61
*b_bp_*(488) Equation (16)	7.16	37.44	2.8 × 10 ^−4^	1.48	48.39	−32.61
*b_bp_*(555) Equation (17)	6.97	36.14	−2.22 × 10 ^−3^	1.48	48.20	−32.52
*b_bp_*(620) Equation (18)	9.95	45.88	2 × 10 ^−5^	1.59	58.90	−37.07

**Table 2:** The relative errors in the approximation.

Step 2. 2-Parameter Model of *IOPs*	Arithmetic Statistic		Logarithmic Statistic			
	Systematic Error	Statistical Error	Systematic Error	Standard Error Factor	Statistical Error	
	<ε> [%]	σ_ε_ [%]	<ε>_g_ [%]	x	σ_ε+_ [%]	σ_ε−_ [%]
*a_ph_*(420) Equation (31)	3.86	28.90	6 × 10 ^−4^	1.32	32.16	−24.33
*a_ph_*(488) Equation (32)	3.62	27.28	−2 × 10 ^−4^	1.32	31.56	−23.99
*a_ph_*(555) Equation (33)	4.21	29.84	1.3 × 10 ^−3^	1.34	33.96	−25.35
*a_ph_*(620) Equation (34)	5.37	33.86	−8 × 10 ^−4^	1.39	39.35	−28.23
*a_d_*(420) Equation (35)	23.5	88.9	−2.2 × 10 ^−3^	1.91	91.1	−47.7
*a_d_*(488) Equation (36)	25.9	91.7	7 × 10 ^−4^	1.99	98.6	−49.7
*a_d_*(555) Equation (37)	36.3	114.1	−1.6 × 10 ^−3^	2.23	123.2	−55.2
*a_d_*(620) Equation (38)	58.0	165.8	2 × 10 ^−4^	2.67	166.7	−62.5
*b_bp_*(420) Equation (39)	8.86	45.31	7 × 10 ^−4^	1.52	52.26	−34.32
*b_bp_*(488) Equation (40)	6.55	38.07	3 × 10 ^−4^	1.44	44.04	−30.58
*b_bp_*(555) Equation (41)	6.02	36.19	−4.4 × 10 ^−3^	1.42	42.04	−29.60
*b_bp_*(620) Equation (42)	8.21	43.85	−1.2 × 10 ^−3^	1.50	50.01	−33.34

**Table 3:** The constants of the five-parameter model of *R_rs_*(*λ_i_*) (Equations (43)–(46)).

λ	C	B	D	K	J	L	*a_w_*	*b_bw_*
420	0.009	0.911	0.337	0.057	0.807	0.750	0.0045	0.0023
488	0.006	0.891	0.827	0.035	0.762	0.903	0.0147	0.0012
555	0.005	0.935	0.977	0.022	0.646	1.157	0.0596	0.0007
620	0.004	0.881	1.230	0.015	0.592	1.542	0.2755	0.0004
λ	F	G	H	M	N	P	*f/Q*	
420	0.827	0.041	0.493	0.077	1.006	0.132	0.07	
488	0.820	0.022	0.824	0.624	1.077	0.485	0.10	
555	0.815	0.011	0.257	1.037	1.072	0.689	0.12	
620	0.926	0.007	0.261	1.488	1.136	0.794	0.13	

**Table 4:** The relative errors in the approximations.

Step 3. 5-Parameter Model of *R_rs_*(*λ_i_*)	Arithmetic Statistic		Logarithmic Statistic			
	Systematic Error	Statistical Error	Systematic Error	Standard Error Factor	Statistical Error	
	<ε> [%]	σ_ε_ [%]	<ε>_g_ [%]	x	σ_ε+_ [%]	σ_ε−_ [%]
*R_rs_*(420)	11.10	34.71	6.13	1.35	35.49	−26.19
*R_rs_*(488)	10.16	34.53	5.46	1.34	34.13	−25.44
*R_rs_*(555)	11.68	37.98	5.,94	1.38	38.25	−27.67
*R_rs_*(620)	13.62	47.52	5.78	1.45	45.12	−31.09

## Data Availability

The data that support the findings of this study are available from the corresponding author upon justified request.
